# Deficient of glycosylation site in the envelop protein attenuated Zika virus replication in mosquito cells

**DOI:** 10.3389/fmicb.2025.1603083

**Published:** 2025-08-25

**Authors:** Wen-Jing Wang, Zi-Han Wang, Jing Li, Sai-Ya Ma, Mei He, Meng-Xuan Liu, Yu-Fei Zhan, Feng Jin, Guosheng Qu, Chunhong Yin, Jie Tong

**Affiliations:** ^1^College of Life Sciences, Hebei University, Baoding, China; ^2^School of Life Sciences and Green Development, Hebei University, Baoding, China; ^3^Shandong Center for Disease Control and Prevention, Infectious Disease Control Institute, Shandong, China; ^4^Shandong Provincial Key Laboratory of Intelligent Monitoring, Early Warning, Prevention and Control for Infectious Diseases, Shandong, China

**Keywords:** Zika virus, E glycoprotein, mosquito cells, ECM signaling pathway, E-dimer

## Abstract

**Introduction:**

The Zika virus (ZIKV) envelope (E) protein is critical for viral replication and host interactions. Although glycosylation of the E protein is known to influence viral infectivity and immune evasion, the specific functional roles of E protein glycosylation in ZIKV infectivity in mosquito cells remain unclear.

**Methods:**

In this study, we generated a deglycosylation mutant ZIKV with a T156I substitution in the E protein and investigated its effects on viral replication and viral-host interactions in mosquito C6/36 cells.

**Results:**

Our results demonstrated that the T156I mutant exhibited attenuated replication compared to the wild-type virus during the early stages (0-24 hours) post-virus infection in mosquito C6/36 cells. This attenuation was associated with reduced E protein expression, which was regulated at the post-transcriptional level. RNA sequencing further revealed that the T156I mutation significantly altered virus-host interactions, particularly affecting the extracellular matrix (ECM) signaling pathway. Notably, several genes involved in the ECM signaling pathway, including THBS1, ITGAL, IL-1A, and CXCL8, were found to inhibit the T156I mutant but not the wild-type ZIKV. Structural analysis and in silico molecular docking suggested that the T156I mutation impaired the stability of the E protein dimer rather than its interactions with neutralizing antibodies.

**Discussion:**

Collectively, these findings provide novel insights into the role of E protein glycosylation in ZIKV infection, and may have significant implications for anti-ZIKV strategies.

## Introduction

Mosquito-borne Zika virus (ZIKV) is primarily transmitted by *Aedes* spp. mosquitoes, especially *Aedes aegypti*. ZIKV is spread through sexual, maternal-to-fetal, and blood transfusions in humans (Gubler et al., 2017). The virus was initially identified in 1947 in Ugandan (Dick et al., 1952), and subsequently, in outbreaks in Africa, Southeast Asia, and the Pacific regions (Musso and Gubler, 2016; Wikan and Smith, 2017; Herrera et al., 2017; Duffy et al., 2009; Musso et al., 2014). In 2015, a new clade of highly virulent ZIKV emerged in South America, particularly Brazil, and rapidly spread to other countries (Cao-Lormeau et al., 2016). Infection with highly virulent ZIKV during pregnancy leads to a variety of congenital malformations known as congenital Zika virus diseases (Faria et al., 2017).

The envelope (E) protein is one of the main surface proteins of ZIKV viral particles and is composed of four domains: the stem/transmembrane domain (E-S/E-TM) that anchors the protein into the viral lipid membrane, and domains I (DI), II (DII), and III (DIII) that constitute the predominant β-strand surface portion of the protein (Cheng et al., 2022). ZIKV has several glycosylation sites in its E protein (e.g., Asn154 and Thr156) that are involved in regulating viral pathogenesis (Fontes-Garfias et al., 2017). These glycosylation sites are also conserved between West Nile virus (WNV) and Japanese encephalitis virus (JEV) (Carbaugh and Lazear, 2020). The glycan loop (named 150-loop) localizes at the interface of the E dimer and facilitates its stability of E dimer (Dai et al., 2016). Nevertheless, several African strains lack glycosylation of the E protein, which contains a 4- to 6-amino-acid deletion in the 150-loop and resulting in the generation of non-glycosylated E proteins (Cheng et al., 2022; Carbaugh et al., 2019; Faye et al., 2014; Annamalai et al., 2017; Frumence et al., 2020). The ablation of glycosylation in the E protein attenuates virus replication and induces slight histological lesions and inflammation in newborn mice (Lunardelli et al., 2022). Therefore, deficiency in the 150-loop may play an important role in the evolution of ZIKV virulence from African strains to American strains.

Intriguingly, the T156I mutation has also been detected in several ZIKV strains isolated from mosquitoes in Africa in the 1970s and the 1980s, which may indicate a pandemic-associated role for T156. It has been demonstrated that T156I mutation inhibits the mid-gut invasion of ZIKV Natal-RGN strain (Bos et al., 2019; Wen et al., 2018). Similarly, the non-glycosylated (N154Q and T156I) Asian strain H/PF/2013 was found to be attenuated in mice, which produced lower viral loads in the serum and brain when inoculated subcutaneously (Mossenta et al., 2017). Moreover, glycosylation of the E protein plays a significant role in ZIKV (Annamalai et al., 2017). The glycan moieties of the E protein interact with host cell receptors, mediating viral infectivity and spreading (Carbaugh and Lazear, 2020; Ishida et al., 2023). Glycosylation of the E protein enhances the infection of cells expressing dendritic cell-specific intercellular adhesion molecule-3-grabbing non-integrin (DC-SIGN) or DC-SIGN-related (DC-SIGNR) (Carbaugh et al., 2019). Since lectin-expressing leukocytes rely on E protein glycosylation for efficient viral uptake, the absence of this modification likely impairs ZIKV entry into these immune cells, contributing to the observed attenuation in mice. However, glycosylation sites on viral surface proteins may have additional roles in enhancing viral infection, including promoting neuro-invasion, influencing viral assembly and release, and evading the host cell’s innate immune responses and other antiviral responses.

In this study, we investigated the effects of the T156I mutation in the E protein of ZIKV on virus replication and host–cell interactions. Substitution of T156 in the virulent strain ZIKV-PRVABC reduced the expression of the E protein and virus titer in the early stage of virus infection. The T156I mutation also had a slight influence on the production of ZIKV viral-like particles in both American and African strains. Further investigation by transcriptomic sequencing indicated a wide range of alterations in mRNA transcription in the T156I mutant virus compared to the wild-type (WT) virus. Understanding the role of glycosylation sites in the ZIKV E protein provides insights into the mechanisms of viral virulence and can inform the development of antiviral strategies and vaccines.

## Results

### T156I mutation delays the replication of ZIKV in mosquito cells

**Figure 1:**
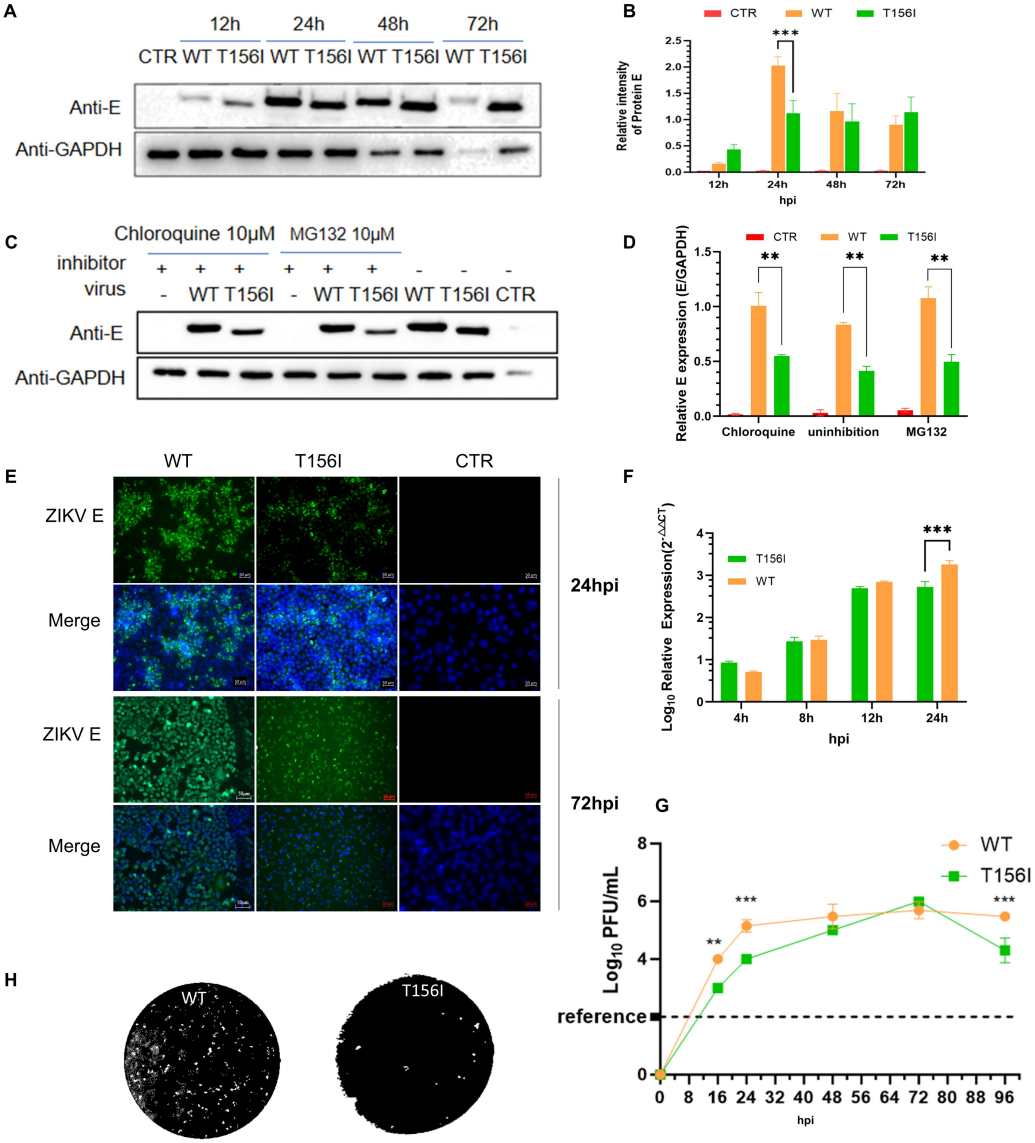
T156I mutation delays the replication of ZIKV in mosquito cells. **(A,B)** The wild type (WT) and T156I mutant (T156I) virus were subjected to infect mosquito C6/36 cells. The whole cell lysates were collected after 12–72 hpi to determine the expression level of E protein using western blotting assay. The GAPDH were used as the house-keeping gene for normalization. The grayscale of the bands in western blotting assay were analyzed by ImageJ software. Asterisks indicate significant differences at 24 hpi between cells infected by WT (orange column) and cells infected by T156I (green column). **(C)** Mosquito cells were infected by WT or T156I virus for 12 hpi and 72 hpi. E protein (green) and cell nucleic (DAPI, blue) were immuno-stained to analyze the expression of E protein. **(D,E)** Chloroquine and MG132 were added to the cells followed by WT or T156I infection. The whole cell lysates were collected after 24 hpi to determine the expression level of E protein using western blotting assay. The GAPDH were used for normalization. **(F,G)** Mosquito cells were infected by WT or T156I. The cell lysates were used to extract the total RNA and subjected to detect the mRNA level of R protein. The supernatants were used to determine the virus titer by plaque-forming assay in VeroE6 cells. **(H)** The morphology of plaques was scanned. All the results represent the mean value ± standard error of the mean pooled from three independent experiments with duplicated samples. Asterisks indicate significant differences between cells infected by WT (orange column) and cells infected by T156I (green column). Statistical analysis was performed with unpaired Student’s *t*-test, *p < 0.01, ^**^p < 0.005, ^***^p < 0.001.

Phylogenetic analysis showed that the T156I mutation occurred in several ZIKV African strains isolated in the 1970s and the 1980s, but not in the later isolates from South America, which potentially indicated that the T156I substitution induced glycosylation deficiency that may originate from the evolution of the virus and is related to viral pathogenesis. Therefore, we first sought to determine whether the change from T156 to I156 could mediate viral virulence. To investigate the effects of the T156I mutation on virus replication, an infectious clone generated from the American strain PRVABC was used to recover the wild-type virus. The T156I mutation was then introduced into the coding sequence of the E protein in the infectious clone. Following the recovery and continuous passage of T156I mutated virus in VeroE6 cells, viral RNA was extracted, and the mutation was confirmed by reverse-transcription and subsequent Sanger sequencing. Previous studies have established that T156 forms part of the conserved N154-X-S/T motif required for N-linked glycosylation of ZIKV E protein; ablation of this glycan disrupts proper E protein folding and ER-Golgi trafficking, resulting in reduced maturation and surface expression (Gong et al., 2018). Therefore, E protein expression was evaluated. The mutated and wild-type viruses were inoculated into C6/36 cells at the same MOI (MOI = 1). The expression of the E protein in the mutated virus (T156I) was compared to that in the wild-type virus. At 24 h post-infection (hpi), the expression level of E protein in the T156I mutant virus was approximately half that of the wild-type virus, indicating a significant decrease. However, at 48- and 72-h post-infection, there were no significant differences in the E protein levels between the mutant and wild-type viruses (Figures 1A,B,E). This indicates that the initial effect of the T156I mutation on E protein expression diminishes over time.

To further determine whether the lower E protein levels in the early stage of virus infection were due to the enhancement of protein degradation, 10 μM proteasome inhibitor (MG132) and 10 μM lysosome inhibitors (Chloroquine, CQ) were used upon virus infection. Upon treatment with MG132 or chloroquine (CQ), there was a slight increase in E protein expression in wild-type-infected cells (approximately 1.3-fold compared to untreated), suggesting minimal involvement of proteasomal or lysosomal degradation under normal conditions (Figures 1C,D). In contrast, the T156I mutant showed no significant change in E protein levels with either inhibitor (less than 1.1-fold). These results indicate that the reduced E protein expression in the mutant is unlikely due to enhanced degradation, and the inhibitory treatments had only a modest effect on wild-type E levels. Therefore, the T156I mutation most likely impairs E protein expression at the level of translation or folding, independent of proteasome or lysosome-mediated clearance.

To determine the virulence of T156I mutated virus, the culture supernatant of virus-infected mosquito cells was collected to test the viral titers by plaque-forming assay, and total RNA was collected to determine the mRNA level of E protein. As shown in Figures 1F,G, in the early stage of virus infection (24 hpi), the T156I mutated virus had a significantly lower titer than the wild-type virus. When the infectious period lasted 48 and 72 h, the titer of the mutated virus reached the equivalent level of the wild-type virus. To further determine the potential effects of viral spread, plaques of wild-type and mutated T156I viruses were compared. As shown in Figure 1H, there are negligible effects on plaque morphology induced by T156I mutation.

### T156I mutation in the E protein alters virus–host interactions

Virus–host interactions are crucial for understanding the pathology, immune responses, and potential therapeutic targets of viral infections. Identifying the host factors involved in the delay of ZIKV infection may provide the groundwork for developing emerging agents to attenuate the spread of ZIKV. RNA-seq was performed to explore differentially expressed genes (DEGs) in wild-type and T156I mutated virus infected cells.

**Figure 2:**
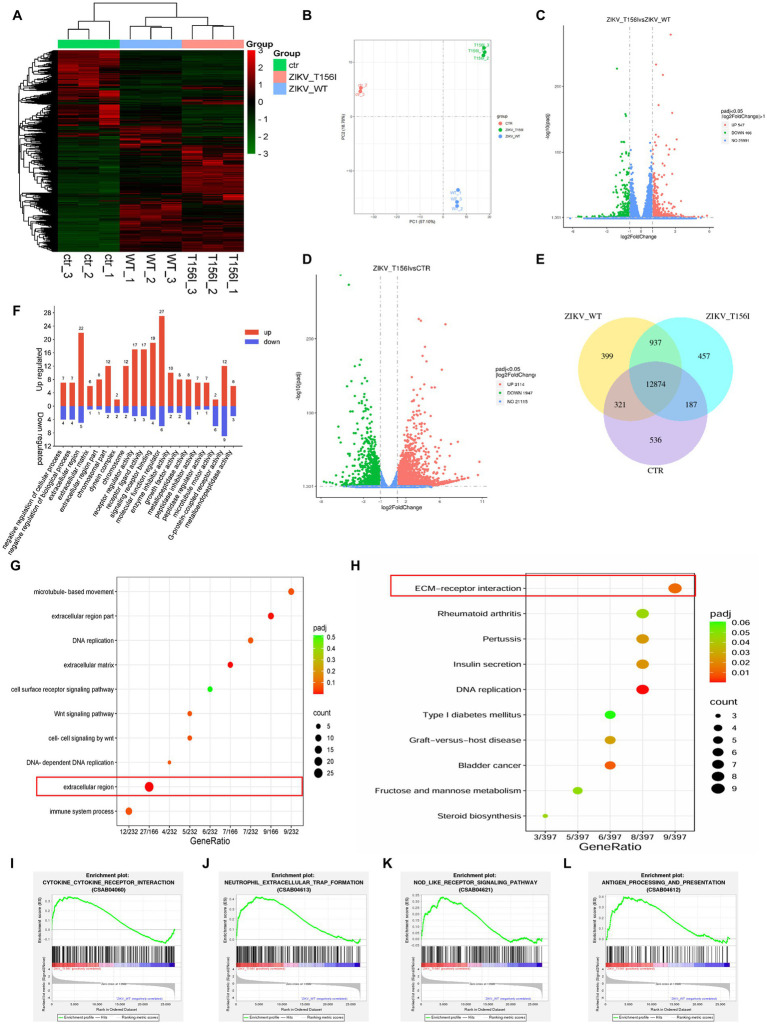
T156I mutation in the E protein alters virus–host interactions. The RNA-seq was applied to explore the differentially expressed genes (DEGs) in wild-type and T156I mutated virus infected cells. **(A)** Heatmap was used to analyze the cluster of DEGs. **(B)** The PCA analysis showed the data within each group indicating satisfactory repeatability. **(C,D)** Volcano maps showed the distribution and quantification of the DEGs between WT and T156I virus infected cells, T156I and uninfected cells, respectively. **(E)** Venn map showed the same and different genes among three different groups, which were WT virus infected cells, T156I infected cells and uninfected cells. **(F)** GO analysis of DEGs between WT and T156I infected cells. **(G,H)** Bubbles map showed the cluster results of DEGs between WT and T156I infected cells based on KEGG and GO database, respectively. **(I–L)** Results of GSEA analysis between WT and T156I infected cells. Cytokine–cytokine receptor interaction (CSAB04060), neutrophil extracellular trap formation (CSAB04613), NOD-like receptor signaling pathway (CSAB04621) and antigen processing and presentation (CSAB04612).

We first performed RNA sequencing to evaluate the general gene expression levels and repeatability in both T156I- and WT-infected cells. As shown in Figures 2A,B, the gene expression levels were generally the same, and the samples within each group also exhibited mutual aggregation, indicating satisfactory repeatability within each group. Hierarchical clustering of differential genes was conducted to confirm the DEGs that were highly expressed in T156I and lowly expressed in WT virus-infected cells. In total, 547 genes were upregulated and 466 genes were downregulated in T156I infected cells compared with the WT virus (Figure 2C), whereas 3,114 genes were upregulated and 1947 genes were downregulated in T156I infected cells compared with non-infected CTR cells (Figure 2D). Co-expression analysis showed that 399 genes were unique to the WT type, and 457 genes were unique to T156I (Figure 2E).

To further analyze the functionality of the (DEGs) based on RNA-seq data, Gene Ontology (GO) and Kyoto Encyclopedia of Genes and Genomes (KEGG) databases were conducted. According to previous reports, the deficiency in glycosylation in envelop proteins may dampen the ability of the virus to adsorb and invade host cells. GO analysis revealed that the upregulated DEGs were mainly associated with the negative regulation of cellular and biological processes in the biological process (BP) term, peptidase regulator activity, and peptidase inhibitor activity in the molecular function (MF) term. Among the CC terms, the DEGs were enriched in the extracellular and chromosomal regions (Figure 2F). Furthermore, the receptor ligand activity, receptor regulator activity, signaling receptor binding, and cell-surface receptor signaling pathways, which may be involved in viral attachment to the host cell surface, were significantly enriched, as revealed by the KEGG pathway analysis (Figures 2G,H). KEGG analysis also showed that the substitution of T156I was related to the host cell immune response upon viral infection. Further analysis by gene set enrichment analysis (GSEA) specifically indicated that the mutation in E protein promotes cytokine–cytokine receptor interaction (CSAB04060) (Figure 2I) and neutrophil extracellular trap formation (CSAB04613) (Figure 2J). Notably, the immune response-related categories such as NOD-like receptor signaling pathway (CSAB04621) (Figure 2K) and antigen processing and presentation (CSAB04612) (Figure 2L) were also significantly upregulated in T156I infected cells comparing to WT infected cells.

Furthermore, KEGG pathway analysis revealed that receptor ligand activity, receptor regulator activity, signaling receptor binding, and cell-surface receptor signaling pathways—potentially involved in viral attachment to the host cell surface—were significantly enriched in cells infected with the wild-type virus but not in those infected with the T156I mutant. This suggests that the loss of glycosylation at position T156 may impair virus–host interactions at the level of receptor engagement and signaling, contributing to the reduced infectivity observed with the mutant.

Overall, RNA-seq revealed a common gene expression diversity between T156I- and WT-infected cells, and the most affected cellular signaling is involved in regulating cell-surface receptors and antiviral immune responses. These results suggest that the T156I mutation may attenuate viral infection and pathogenicity by dampening viral attachment to host cells and stimulating more intensive immune responses.

### Genes in extracellular matrix signaling pathway mediate the attenuation of virus infection which induced by E protein glycosylation deficiency

**Figure 3:**
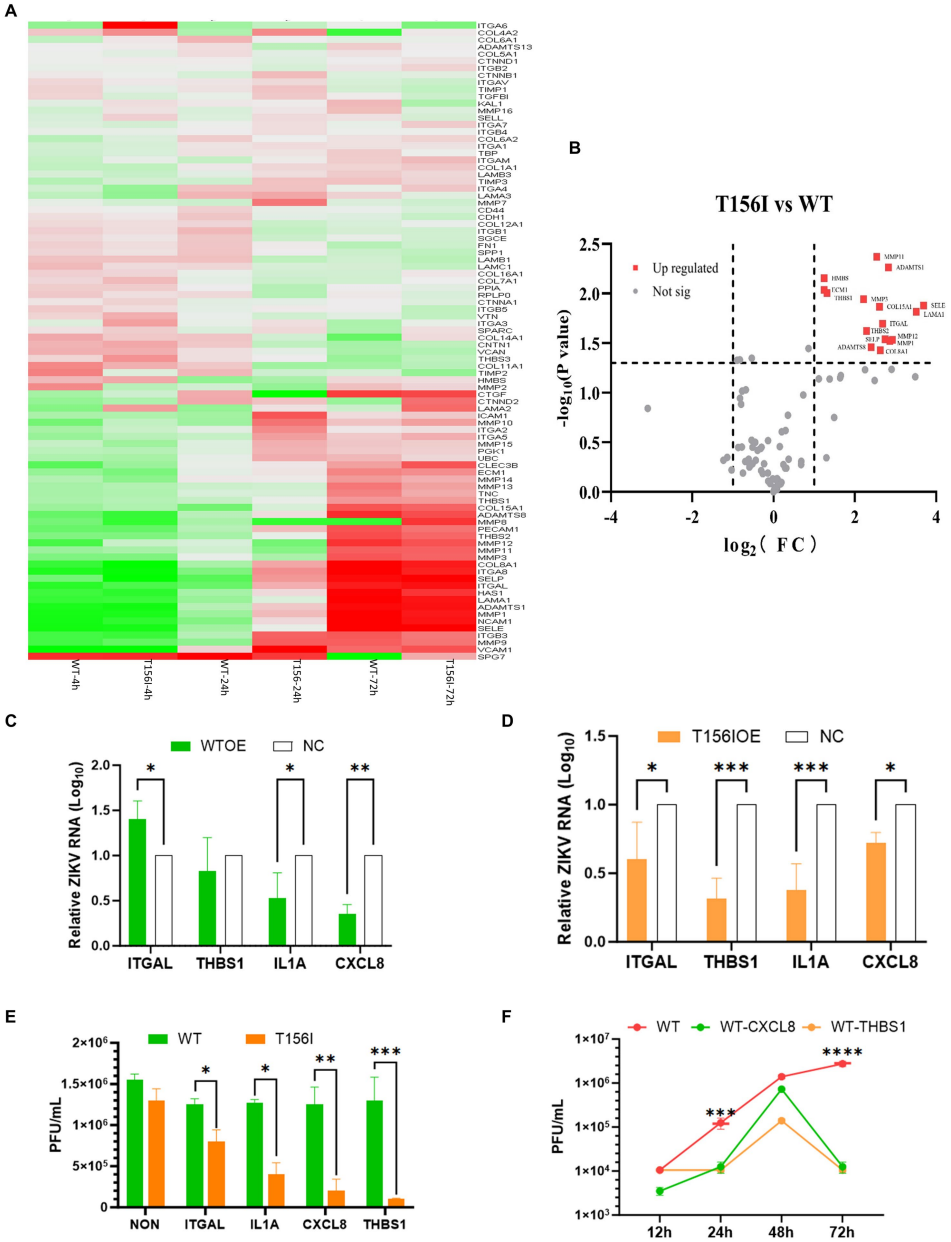
Genes in extracellular matrix (ECM) signaling pathway mediate the attenuation of virus infection which induced by E protein glycosylation deficiency. **(A)** Eighty-four genes involving in ECM pathway were detected by PCR array at 4-, 24- and 72-h post-infection. The differential gene expression at various time points following WT and T156I virus infection was analyzed using a clustered heatmap of the PCR array results. **(B)** Host genes with significant differential expression and substantial changes in expression levels are highlighted with red dots, including ITHAL and THBS1, among others. **(C,D)** The WT virus an T156I virus were inoculated with empty vector transfected control cells (NC) and overexpression (OE) cells after 24 h post transfection, respectively. The supernatants and the total RNA of the viral infected-culture cells were collected at another 24 h post-infection. The mRNA level of E protein and virus titer were determined by qRT-PCR and plaque-forming assay. **(E)** The viral replication kinetics were detected after 12–72 hpi of WT virus in OE cells or NC cells. The viral titers were determined by plaque forming assay. **(F)** The viral titers were detected after 72 hpi of WT infected cells, either overexpressed of ITGAL, IL-1A, CXCL8, and THBS1 or only transfected with empty vectors. All the results represent the mean value ± standard error of the mean pooled from three independent experiments with duplicated samples. *p < 0.01, ^**^p < 0.005, ^***^p < 0.001.

In *Ifnar1*^−/−^ mice, deletion of the conserved N154-X-S/T glycosylation motif in the ZIKV E protein completely abolished infection of dendritic cells expressing DC-SIGN or DC-SIGNR, demonstrating that E-glycan engagement of these C-type lectins is essential for viral entry into lectin-positive leukocytes (Carbaugh et al., 2019). Among the various host factors that may be involved in viral attachment and entry, the extracellular matrix (ECM) signaling pathway may facilitate viral infections by mediating the early stages of the viral life cycle. Certain viruses bind to ECM proteins, such as heparan sulfate or integrins on the cell surface, which act as co-receptors or attachment factors that aid in viral entry. Moreover, the ECM and its associated signaling pathways regulate cell migration. Viruses can manipulate these pathways to enhance the migration of infected cells, thereby promoting the spread of the virus within tissues and other parts of the body. As showed in our RNA-seq-derived KEGG pathway enrichment (Figures 2G,H), which identified ECM-receptor interaction as the most significantly altered pathway in wild-type-infected cells (adjusted *p*-value = 4.3 × 10^−5^), and is supported by prior reports demonstrating that ECM remodeling modulates flavivirus entry and dissemination. Therefore, we examined the gene expression of ECM signaling pathways in T156I infected cells. Eighty-four genes involving in the ECM pathway were detected by PCR at 4, 24- and 72-h post-infection. Differential gene expression at various time points following WT and T156I virus infection was analyzed using a clustered heatmap of the PCR array results. Accordingly, nearly half of the genes in the ECM signaling pathway were differentially expressed in WT and T156I virus-infected cells at 24 hpi (Figure 3A), whereas the different genes showed non-significant changes at 4 and 72 hpi. These differences were also observed in RNA sequencing. These findings indicated that the impact of the T156I mutation on viral replication showed dynamic changes. Specifically, no significant differences were observed in the early (4 hpi) and late (72 hpi) stages of infection, whereas a marked divergence was evident at 24 hpi.

To further investigate the host genes within the ECM signaling pathway that significantly influence viral replication, we conducted a comprehensive analysis based on both the magnitude of differential expression and changes in gene copy number. As shown in Figure 3B, host genes with significant differential expression and substantial changes in expression levels are highlighted with red dots, including ITHAL and THBS1. ITGAL encodes integrin alpha L, a protein involved in immune cell adhesion and migration, which can influence antiviral responses and recruitment of immune cells to infection sites. THBS1 encodes thrombospondin-1, a glycoprotein that modulates cell–cell and cell–matrix interactions, often enhancing viral entry or replication by affecting the extracellular matrix. Based on the integrated RNA-seq and PCR array results, we selected other 2 genes for further investigation to determine their effects on the replication of both the T156I and WT viruses. IL-1A encodes interleukin-1 alpha, a pro-inflammatory cytokine that stimulates immune responses to viral infection and contributes to inflammation and immune regulation. CXCL8 encodes interleukin-8, a chemokine that attracts neutrophils to sites of infection and plays a role in inflammation and immune defenses against viruses.

The coding sequences of these four genes were cloned into the pcDNA3.1 vectors. After confirmation of the correct insertion by Sanger sequencing, the genes were overexpressed in VeroE6 cells by transient transfection prior to virus infection. At 24 hpi, the mRNA levels of these genes in overexpression (OE) cells were markedly higher than those in non-transfected control cells (data not shown), confirming successful overexpression. Then, the WT virus and T156I virus were inoculated into NC and OE cells 24 h post-transfection. The supernatants and total RNA of the virus-infected cultured cells were collected at 24 h post-infection. The mRNA levels of E protein and virus titer were determined using qRT-PCR and a plaque-forming assay. As shown in Figure 3D, the mRNA level of E protein decreased in all the OE cells compared to NC cells in T156I infected cells. Consistently, the viral titer also showed a significant decline in OE cells infected with T156I virus (Figure 3F). These results suggested the potential antiviral functions of ITGAL, THBS1, IL-1A, and CXCL8. These hypotheses were further tested in the WT virus-infected cells. As speculated, the overexpression of IL-1A and CXCL8 attenuated E protein expression and viral replication (Figures 3C,F). However, THBS1 and ITGAL only slightly reduced even increased the virus mRNA level in WT-infected cells (Figure 3C). Consistently, the viral titer in T156I virus-infected cells were significantly lower than WT virus-infected cells upon overexpression of ITGAL, THBS1, IL-1A, and CXCL8 (Figure 3E). This differential response implied that the T156I mutation may render the virus more sensitive to the antiviral effects of certain host genes. To further investigate the impact of THBS1 and ITGAL on WT virus replication, we overexpressed these two genes in Vero E6 cells prior to viral infection and monitored viral replication levels at 12, 24, 48, and 72 hpi. The results indicated that at 24 and 72 hpi, cells overexpressing these genes showed a significant decrease in viral replication compared with cells without overexpression; however, this difference was somewhat attenuated at 48 hpi (Figure 3F). Therefore, THBS1 and ITGAL also have potential antiviral abilities in WT-infected cells, depending on the virus replication kinetics. Overall, the overexpression assay indicated the antiviral functions of THBS1, ITGAL, IL-1A, and CXCL8.

### T156I mutation potentially weaken the stability of E-dimer but not antibody receptor binding affinity

**Figure 4:**
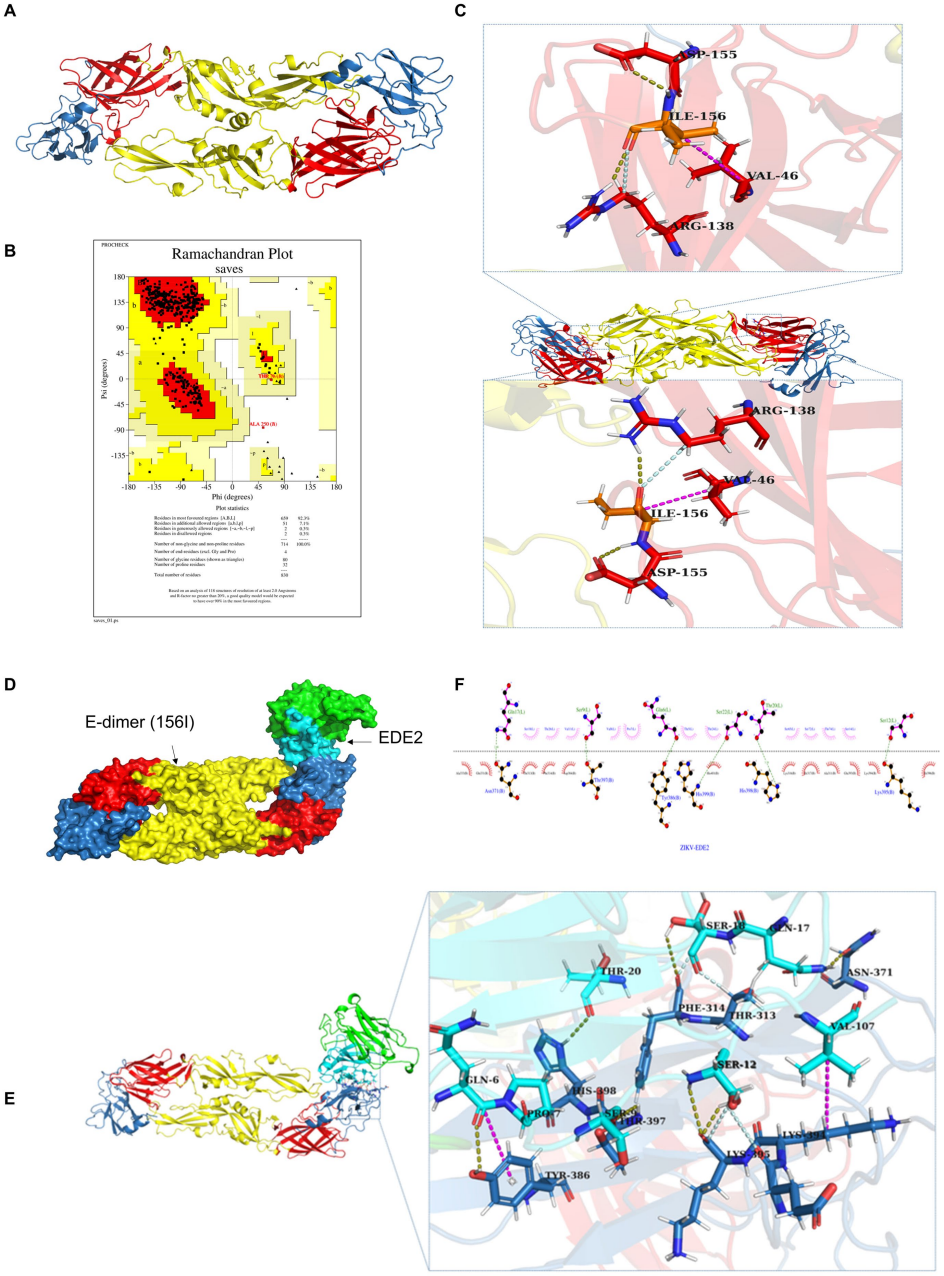
T156I mutation potentially weaken the stability of E-dimer but not antibody receptor binding affinity **(A)** The molecular simulation of the T156I mutation on the structure of ZIKV E protein. The red, yellow, and blue colors represent the domain I, domain II and domain III of the E protein which mediate the formation of E-dimer. **(B)** The protein model was evaluated using Ramachandran plot analysis. The results indicate that 92.3% of the amino acids are located in the most favorable region, 7.1% in additional allowed regions, 0.3% in generously allowed regions, and 0.3% in disallowed regions-totaling 100%, which confirming the reliability of the E protein structural model. **(C)** The potential interaction patterns at the 156I residue with neighboring amino acids. Yellow stick represents the hydrogen bonds, pink stick showed the weak C–H bonds, and the gray stick represent the two sets of hydrophobic interactions with surrounding residues. **(D)** The HDCOK program was used to perform a global docking simulation of the binding interface and interaction mode between the 156I mutant E protein and the ZIKV neutralizing antibody EDE2. **(E)** Revealed that the mutant complex forms seven sets of hydrogen bonds (gray), four sets of weak C–H bonds (yellow), and two sets of hydrophobic interactions (pink). **(F)** The potential interactions of I156 with surrounding residues in 2D analysis.

In addition to affecting the glycosylation of E protein, the substitution of threonine (T) with isoleucine (I) may also alter the structure of E protein and, consequently, its function. Therefore, we employed molecular simulation to analyze the impact of the T156I mutation on the structure of the E protein and aimed to elucidate a possible mechanism by which this mutation influences viral replication. As shown in Figure 4A, the red, yellow, and blue colors represent domains I, II, and III of the E protein, which mediate the formation of the E-dimer. The protein model was first evaluated using Ramachandran plot analysis. The results indicated that 92.3% of the amino acids were located in the most favorable region, 7.1% in additional allowed regions, 0.3% in generously allowed regions, and 0.3% in disallowed regions-totaling 100%, which confirmed the reliability of the E protein structural model (Figure 4B). To further study the effects of the T156I mutation on the stability of the E-dimer, we examined the potential interaction patterns of the 156I residue with neighboring amino acids. As shown in Figure 4C, the mutated residue 156I formed four sets of hydrogen bonds, two sets of weak C–H bonds, and two sets of hydrophobic interactions with the surrounding residues (Table 1). Compared to the wild-type E protein, which contains 156 T, both the number of hydrogen bonds and hydrophobic interactions were reduced. Therefore, we hypothesized that the T156I mutation compromises the stability of the E protein dimer.

**Table 1:** The interactions between I156 with surrounding amino acid residues in E-dimer.

Name	Category
A: ARG138: HH11—A: ILE156: O	Hydrogen bond
A: ILE156: HN—A: ASP155: OD1	Hydrogen bond
B: ARG138: HH11—B: ILE156: O	Hydrogen bond
B: ILE156: HN—B: ASP155: OD1	Hydrogen bond
A: ARG138: CD—A: ILE156: O	Carbon hydrogen bond
B: ARG138: CD—B: ILE156: O	Carbon hydrogen bond
A: VAL46—A: ILE156	Hydrophobic
B: VAL46—B: ILE156	Hydrophobic

E protein is the most important surface protein on the ZIKV envelope. Previous studies have indicated that it may play a critical role in mediating interactions between the virus and neutralizing antibodies. Therefore, to investigate the potential effect of the T156I mutation of E protein in the interaction between ZIKV and neutralizing antibodies, we employed the HDCOK program to perform a global docking simulation of the binding interface and interaction mode between the 156I mutant E protein and the ZIKV neutralizing antibody EDE2. The structure with the best docking score was selected as the standard result for further analysis. The docking score was calculated using the ITScorePP (or ITScorePR) iterative scoring function. The optimal docking structure is shown in Figure 3D, with a docking score of −268.29 and a confidence score of 0.9142, indicating that the complex model is highly reliable. Further analysis of the intermolecular interactions between the 156I mutant E protein and EDE2, as presented in Table 2 and Figures 4E,F, revealed that the mutant complex formed seven sets of hydrogen bonds, four sets of weak C–H bonds, and two sets of hydrophobic interactions. The presence of these interactions may further strengthen the binding affinity of ZIKV E protein to EDE2, as well as the possibility of spontaneous binding. However, this docking result did not show any significant difference compared with the interaction between the wild-type E protein and EDE2, implying that the T156I mutation may not have a significant impact on the binding between ZIKV and the neutralizing antibody.

**Table 2:** The interactions between I156 with surrounding amino acid residues in E-dimer with neutralization antibody complex.

Name	Category
ZIKV-B: TYR386: HH—EDE2-L: GLN6: O	Hydrogen bond
ZIKV-B: THR397: HN—EDE2-L: SER9: O	Hydrogen bond
ZIKV-B: HIS398: HD1—EDE2-L: THR20: O	Hydrogen bond
EDE2-L: SER12: HN1—ZIKV-B: LYS395: O	Hydrogen bond
EDE2-L: SER12: HG—ZIKV-B: LYS395: O	Hydrogen bond
EDE2-L: GLN17: HE22—ZIKV-B: ASN371: OD1	Hydrogen bond
EDE2-L: SER18: HG: B—ZIKV-B: PHE314: O	Hydrogen bond
ZIKV-B: THR313: HB—EDE2-L: SER18: O	Carbon hydrogen bond
EDE2-L: SER12: HB2—ZIKV-B: GLU393: O	Carbon hydrogen bond
EDE2-L: SER12: HB2—ZIKV-B: LYS395: O	Carbon hydrogen bond
EDE2-L: SER18: HB1—ZIKV-B: PHE314: O	Carbon hydrogen bond
ZIKV-B: LYS394—EDE2-L: VAL107	Hydrophobic
ZIKV-B: TYR386—EDE2-L: PRO7	Hydrophobic

## Discussion

Glycoproteins are essential for viral infection, facilitating host cell entry, membrane fusion, immune evasion, and viral assembly. They mediate binding to host receptors (e.g., SARS-CoV-2 spike to ACE2, HIV gp120 to CD4), determine viral tropism, and can shield viral epitopes with glycans to escape immune detection (Walls et al., 2020; Wei and Yu, 2016). In addition, glycoproteins contribute to virion assembly and budding, and are key targets for neutralizing antibodies and antiviral therapies, making them central to viral pathogenesis and vaccine design (Mifsud et al., 2024). The glycosylation status of viral proteins may represent an evolutionary trade-off between optimal replication in mosquito versus human cells. In mosquito cells, the glycosylation machinery is less complex, and excessive or mammalian-type glycosylation can impair viral protein processing or virion assembly. Conversely, in human cells, glycosylation often enhances viral protein stability, immune evasion, and infectivity. For example, the loss of glycosylation at specific sites may improve replication efficiency in mosquito cells by streamlining protein folding, but simultaneously reduce viral fitness in mammalian hosts due to increased immune recognition or impaired receptor binding. This trade-off suggests that viruses must balance glycosylation to maintain transmission efficiency between vector and host, highlighting its critical role in cross-species adaptation.

As an arbovirus, ZIKV requires vertebrate and invertebrate hosts to complete its transmission cycle (Petersen et al., 2016). The interaction between ZIKV and mosquito host cells is critical for viral survival, replication, and dissemination within the vector (Boyer et al., 2018). Studying these responses can uncover the mechanisms that allow ZIKV to persist in mosquitoes without causing cytopathogenic effects, which are distinct from their interactions with mammalian cells. Investigating host cell responses can help identify key molecular pathways that facilitate ZIKV replication. For example, as indicated in our study, ECM pathways may contribute to immune evasion in mosquitoes. Regulates the virus’s ability to cross the midgut barrier, reach the salivary glands, and ensure efficient transmission to vertebrate hosts. Moreover, by understanding how mosquito cells respond to ZIKV infection, some key genes such as ITGAL, CXCL8, and THBS1 may be targeted to disrupt the virus’s replication or transmission. Gene editing technologies, such as CRISPR, can be employed to knock out mosquito genes essential for ZIKV survival. Similarly, RNA-interference (RNAi)-based approaches have been developed to suppress ZIKV replication in mosquitoes.

ECM-related host genes may influence viral replication through several pathways. Structural components such as collagen, laminin, and fibronectin contribute to the physical integrity and signaling landscape of the extracellular environment, which in turn modulates cell adhesion, migration, and immune activation—factors that are critical during viral infection (Borges-Vélez et al., 2022; Chen et al., 2013). Certain ECM proteins can regulate the availability of growth factors and cytokines, thereby indirectly influencing viral replication by shaping the host’s antiviral response (Tomlin and Piccinini, 2018; Liu et al., 2024). In addition, ECM remodeling enzymes, such as matrix metalloproteinases (MMPs), can alter tissue architecture and facilitate viral dissemination by breaking down physical barriers (Bhardwaj and Singh, 2023). Some viruses may exploit ECM-integrin signaling to promote entry or enhance intracellular signaling conducive to replication (Liu et al., 2024). Therefore, dysregulation or modification of ECM-related genes during infection could either restrict or facilitate viral replication depending on the context and virus–host interactions. ECM pathway also exhibits striking tissue-specific dynamics in monkeys versus mosquitoes that likely influence arbovirus infection outcomes: in primates like rhesus or cynomolgus macaques, ECM components such as fibronectin, collagen, proteoglycans and associated enzymes (e.g., MMPs/TIMPs) actively signal to innate immune cells, modulating macrophage activation, phagocytosis and cytokine production, and undergo rapid remodeling during viral invasion to orchestrate tissue repair and inflammation (Stavolone and Lionetti, 2017); in contrast, mosquito vectors lack a comparable multicellular ECM in their midgut or salivary glands, relying instead on cell-intrinsic receptors and virus–vector interactions mediated through RNA elements like the 3′ untranslated region, which profoundly influence replication efficiency in mosquito cells but not in mammalian cells (Chen et al., 2013). These mechanistic differences suggest ECM remodeling in primate hosts not only restricts viral dissemination via immune modulation but may also paradoxically create niches facilitating cell-to-cell spread, whereas mosquitoes support transmission through alternative molecular pathways, underscoring ECM’s dual role in shaping pathogen transmission dynamics.

ZIKV’s ability to adapt to mosquito host environments may influence its evolutionary trajectory, transmission dynamics, and virulence in humans (Tramonte and Christofferson, 2019). Exploring how mosquito cells respond to ZIKV infection can provide insights into the selective pressures shaping the virus, which may inform predictions about future outbreaks. Detailed knowledge of the mosquito response to ZIKV infection can also guide the development of transgenic mosquito lines that are resistant to ZIKV or other arboviruses. Such efforts can significantly reduce the transmission potential of the virus. Effective control of arbovirus transmission at the vector level can prevent human infection, particularly in regions with high mosquito densities and endemic arboviruses. By focusing on mosquito host responses, we not only gain fundamental knowledge of arbovirus biology, but also open pathways for novel vector control strategies and reduce the burden of ZIKV-related diseases in human populations.

Furthermore, understanding host cell responses is an integral part of a broader strategy for mitigating ZIKV outbreaks. Analyzing the differences in host cell responses towards T156I mutated and WT virus infections is important. When the same experiments were performed with WT and T156I viruses, only IL-1A and CXCL8 maintained a strong inhibitory effect, whereas THBS1 and ITGAL showed only minor effects. This differential response implied that the T156I mutation may render the virus more sensitive to the antiviral effects of certain host factors. Interestingly, the 156I mutation occurred in ancient African strains but not in pandemic Asian strains (Haddow et al., 2012). Therefore, the 156I to 156 T mutation may also be an important pandemic-associated mutation in the E protein, which enhances viral pathogenicity by dampening the sensitivity of viral responses to host antiviral factors. Our results also point to the potential of boosting the IL-1A and CXCL8 pathways as antiviral strategies in ZIKV infection. At the same time, our results highlight that not all host molecules (such as THBS1 and ITGAL) uniformly affect viral replication, suggesting that their antiviral roles might depend on specific viral mutations or interaction contexts.

IL-1A is an early-response pro-inflammatory cytokine that is rapidly up-regulated during many acute viral infections, including influenza A and dengue virus, where it amplifies local inflammation and recruits neutrophils and monocytes to sites of infection (Dinarello, 2011). In dengue, IL-1A release by infected macrophages contributes both to endothelial activation and to vascular leakage (Pan et al., 2019). In ZIKV infection, elevated IL-1A (as we observed) may similarly drive blood–brain barrier permeability and enhance glial activation, thereby promoting neuroinflammation and facilitating viral entry into the central nervous system via disruption of tight junctions.

CXCL8 (IL-8) is a potent neutrophil chemoattractant whose expression is induced by viruses such as RSV and hepatitis C virus to shape early innate responses (Nuriev and Johansson, 2019). CXCL8 also has been shown to enhance viral replication indirectly by promoting oxidative stress and paracrine cytokine production (Kong et al., 2008). In our ZIKV model, up-regulation of CXCL8 could attract neutrophils and monocytes that ZIKV can infect, creating additional cellular reservoirs and amplifying local viral burden.

THBS1 (Thrombospondin-1) is a matricellular glycoprotein that modulates cell–matrix interactions, angiogenesis, and TGF-β activation. In HIV and CMV infection, THBS1 expression exacerbates tissue remodeling and fibrosis, contributing to chronic organ damage (Crombie et al., 1998; Khaiboullina et al., 2016). In the context of ZIKV, THBS1 up-regulation may participate in ECM remodeling that both enhances viral dissemination through basement membrane degradation and activates latent TGF-β signaling, which has been implicated in immunosuppression and viral persistence.

ITGAL (CD11a) is a component of the LFA-1 integrin complex critical for leukocyte adhesion, extravasation, and immune synapse formation. In West Nile virus, LFA-1-mediated trafficking of infected monocytes into the brain is a key step in neuropathogenesis (Lim and Murphy, 2011; Haynes et al., 1989). Elevated ITGAL in ZIKV-infected cells may similarly facilitate infected cell adhesion to the endothelium and trans-endothelial migration of leukocytes harboring virus, thereby promoting neuroinvasion.

Together, these four factors suggest a feed-forward loop in ZIKV infection whereby ECM remodeling (THBS1) and enhanced leukocyte trafficking (ITGAL), coupled with pro-inflammatory chemokine (CXCL8) and cytokine (IL-1A) release, synergize to both recruit additional susceptible cells and compromise barrier integrity, amplifying viral spread and immunopathology. Targeting one or more of these pathways may therefore limit ZIKV dissemination and tissue damage.

ECM-related host genes were tested in Vero-E6 cells because these cells provide a well-characterized and highly permissive mammalian system that allows for robust detection of viral replication and host gene manipulation. Although mosquito cells are central to the transmission cycle, they possess limited ECM components and distinct glycosylation and immune signaling pathways compared to mammalian cells. Since the functional consequences of ECM gene modulation are more readily interpretable in the context of a complex ECM network, Vero-E6 cells serve as a suitable model to dissect the role of host ECM genes in supporting or restricting viral replication. Future studies using mosquito-derived cells will be needed to directly assess the vector-specific relevance of these findings.

The T156I mutation affects ZIKV adsorption and/or invasion efficiency may derived from at least three aspects. The T156I mutant exhibited significantly delayed replication kinetics (Figure 1G), with reduced viral titers at early timepoints (12–24 hpi), which is a phenotype inconsistent with major adsorption defects. As demonstrated in previous study, adsorption-impaired mutants typically show near-normal replication once intracellular infection is established, whereas our mutant shows sustained impairment. We observed altered ECM remodeling (Figures 2, 3) and upregulation of ECM1 (a key invasion regulator) in T156I-infected cells. This parallels findings in previous study where glycosylation-deficient flaviviruses dysregulated ECM pathways without altered adsorption efficiency. Glycosylation at T156 (N154 in some strains) locates near Domain II fusion loop. Literature confirms that glycosylation at this site stabilizes E protein dimers post-fusion, explaining our observed defects in E-dimer stability rather than neutralizing antibody attachment.

In summary, by demonstrating that mutations at the glycosylation site of ZIKV E proteins (T156I) affect viral replication in mosquito cells, our study highlights a potential molecular determinant that may influence vector competence. Glycosylation of envelope proteins has been implicated in the efficiency of viral entry into mosquito midgut cells and subsequent replication. Identifying T156 as a critical site suggests that even subtle modifications in viral glycosylation can modulate the ability of the virus to be transmitted by its vector, potentially affecting epidemic dynamics. If the T156 mutation is associated with altered viral replication in mosquitoes, it may contribute to differences in viral load and transmission efficiency. This could help explain the variations in epidemic severity, making this glycosylation site a potential marker for assessing outbreak risk. Understanding such determinants is essential for developing predictive models of virus spread and informing public health interventions. In addition to viral factors, we identified several key host genes and associated pathways that appear to have antiviral effects. This research opens new avenues for novel therapeutic strategies by elucidating how host factors counteract viral replication. The combined analysis of viral mutations and host gene responses provides a comprehensive picture of virus–host interactions. This dual focus not only deepens our understanding of ZIKV pathogenesis but also identifies targets for intervention at both the viral and host levels. Such integrative insights are invaluable for designing strategies that could mitigate ZIKV outbreaks, whether through vector control measures or bolstering host immunity. Collectively, these findings offer new perspectives on controlling ZIKV transmission and improving public health responses to future outbreaks.

## Materials and methods

### Cells and viruses

Vero-E6 cells (CRL1586, ATCC), were cultured in Dulbecco’s modified Eagle’s medium (DMEM; Gibco) containing 10% fetal bovine serum (FBS; Sigma) and 1% penicillin/streptomycin (P/S, Thermo Fisher Scientific) at 37 °C with 5% CO_2_. Mosquito C6/36 cells (CRL1660, ATCC) were cultured in Dulbecco’s modified Eagle’s medium (DMEM; Gibco) containing 10% fetal bovine serum (FBS; Sigma) and 1% penicillin/streptomycin (P/S, Thermo Fisher Scientific) at 30 °C with 8% CO_2_.

ZIKV strain PRVABC59 (GenBank: KU501215) was recovered from an infectious cDNA clone. The T156I mutation was introduced into an infectious clone by site-specific mutagenesis. The sequence-verified infectious clone plasmids were linearized with M*lu* I (New England Biolabs, R3198S) and purified by phenol-chloroform extraction (P.C.I). *In vitro*-transcribed viral RNA was prepared using a HiScribe^®^ T7 ARCA mRNA Kit (New England Biolabs, E2065S) and purified by phenol-chloroform extraction (P.C.I). The viral mRNA was then transfected into Vero-E6 cells by electroporation using Gene Pulser X cell^™^ (Bio-Rad, 165-2660). The supernatants were subsequently collected at 5–8 days post-transfection, when the cytopathic effect reached 40%. The genomic RNA of the recovered virus was extracted and sequenced using RT-PCR to determine whether the substitution occurred.

### Plaque assay

The samples were serially diluted (10×) in infectious medium, and 1 mL diluted virus was added to a monolayer of Vero-E6 cells in 12-well plates. After 1-h for the attachment of virus attachment, the supernatants were discarded and the cells were washed twice with phosphate-buffered saline (PBS). Then, 1.5 mL of culture medium containing 1% methyl cellulose (Sigma, #9004-67-5) was added to each well. The cells were subsequently incubated at 37 °C and 5% CO_2_ for several days. To calculate the virus titer, a crystal violet solution was added to stain and count the virus plaques. All experiments were repeated in three times with duplicate samples.

### RNA extraction

Viral RNAs or the total RNA of infected cells was extracted using TRIzol^™^ Reagent (Solarbio, 15596-018), which was further purified by phenol-chloroform extraction (P.C.I). The concentration and purity of the RNA samples were determined using a Nanodrop 2000. RNA integrity was assessed by agarose gel electrophoresis, and the RIN value was determined using Agilent 2100 to ensure quality compliance for subsequent procedures.

### Transcriptome high-throughput sequencing

#### Library preparation and quality control

The integrity of the RNA samples was assessed using the RNA Nano 6000 Assay Kit on the Bioanalyzer 2100 system (Agilent Technologies, CA, United States). Total RNA was used as the input for sample preparation. mRNA was purified from total RNA using poly T oligo-attached magnetic beads. Fragmentation was carried out using divalent cations at elevated temperatures in a first-strand synthesis reaction buffer. First-strand cDNA was synthesized using random hexamer primers and M-MLV reverse transcriptase (RNase H^−^). Second-strand DNA synthesis was subsequently performed using DNA polymerase I and RNase H. Remaining overhangs were converted into blunt ends via exonuclease/polymerases. After adenylation of the 3′ ends of the DNA fragments, the adaptor with a hairpin loop structure was ligated to prepare for hybridization. To select cDNA fragments preferentially (370–420 bp in length), the library fragments were purified with the AMPure XP system. PCR was then performed using Phusion High-Fidelity DNA polymerase, universal PCR primers, and the index primer. PCR products were purified with the AMPure XP system, and library quality was assessed on the Agilent Bioanalyzer 2100 system.

Clustering of the index-coded samples was performed on a cBot Cluster Generation System using the TruSeq PE Cluster Kit v3-cBot-HS (Illumina) according to the manufacturer’s instructions. After cluster generation, the library preparations were sequenced on an Illumina NovaSeq platform and 150 bp paired-end reads were generated.

#### Data collection and analysis

Raw data (raw reads) were processed using the fastp software. Clean data (clean reads) were obtained by removing reads containing adapters, poly-N, and low-quality reads from raw data. At the same time, the Q20, Q30, and GC contents of the clean data were calculated. All downstream analyses were based on high-quality clean data.

Reference genome and gene model annotation files were downloaded from the NCBI database. The index of the reference genome was built using Hisat2 v2.0.5, and clean paired-end reads were aligned to the reference genome using Hisat2 v2.0.5. We selected Hisat2 as the mapping tool because Hisat2 can generate a database of splice junctions based on the gene model annotation file and thus provide a better mapping result than other non-splice mapping tools.

#### Quantification of gene expression level

Counts v1.5.0-p3 were used to count the read numbers mapped to each gene, and the expected number of fragments per kilobase of transcript sequence per million (FPKM) of each gene was calculated based on the length of the gene and the read count mapped to this gene. FPKM considers the effect of sequencing depth and gene length for read counts simultaneously, and is currently the most commonly used method for estimating gene expression levels.

#### Differentially expressed genes analysis

Differential expression analysis of the two groups was performed using the DESeq2 R package (version 1.20.0). The resulting *p*-values were adjusted using Benjamini and Hochberg’s approach to control the false discovery rate. Genes with an adjusted *p*-value ≤0.05 were found by DESeq2 and assigned as differentially expressed genes.

#### GO and KEGG enrichment analysis of differentially expressed genes

GO enrichment analysis of differentially expressed genes was performed using the Cluster Profiler R package, in which the gene length bias was corrected. GO terms with corrected *p*-values less than 0.05 were considered significantly enriched. Similarly, the cluster profiler R package was applied to test the statistical enrichment of differentially expressed genes in the KEGG pathways.

#### Gene set enrichment analysis

Gene set enrichment analysis (GSEA) is a computational approach to determine whether a predefined gene set can show a significant consistent difference between two biological states. The genes were ranked according to the degree of differential expression in the two samples, and the predefined gene set was tested to determine if they were enriched at the top or bottom of the list. We used the local version of the GSEA analysis tool, and the GO or KEGG dataset was used for GSEA independently.

### PCR array

A549 cells were inoculated with viruses at an MOI of 0.1, and total RNA was harvested 48 h post-infection (hpi). To investigate the gene expression profile, a PCR array assay was performed using an extracellular matrix and adhesion molecule PCR array plate (Wcgene Biotech, WC-MRNA0057-H). Total RNA was extracted using TRIzol^™^ Reagent (Solarbio, 15596-018) and purified by phenol-chloroform extraction (P.C.I). RNA concentration and purity were measured using Nanodrop 2000, and RNA integrity was assessed by agarose gel electrophoresis. cDNA was synthesized using the StarScript III RT Kit (GenStar, A232-10), according to the manufacturer’s instructions. All experiments were repeated in three times with duplicate samples.

### Transient overexpression

The ectodomains of the WT and mutant viruses were inserted into the pLVX vector for transformation into 293 T cells. The pLVX vector is a lentiviral expression vector with a constitutive CMV promoter and puromycin resistance marker that expresses the recombinant zsGreen protein. Positive clones were identified by PCR and DNA sequencing and then transformed into DH5α cells to obtain recombinant plasmids. For transient overexpression of the ZIKV envelope protein, cells were transfected using Lipofectamine 2000 (Invitrogen, 11668) according to the manufacturer’s instructions. After 24–48 h of transfection, the cells were harvested for further analysis. The efficiency of overexpression was verified by western blotting as described below. All experiments were repeated in three times with duplicate samples.

### Western blotting

Western blotting was performed to detect the expression levels of the target proteins. Cells were lysed in RIPA buffer (Epizyme, PC104), and the protein concentration was determined using a Modified BCA Protein Assay Kit (Sangon Biotech, C503051-0500). Equal amounts of protein (usually 30–50 μg) were separated by SDS-PAGE and transferred onto a PVDF membrane (Millipore, IPVH00010). The membrane was blocked with 5% non-fat milk in TBST for 1 h at room temperature, followed by incubation with primary antibody against Zika virus (ZIKV) envelope protein (SinoBiological, 40543-R029) overnight at 4 °C. After washing, the membrane was incubated with an HRP-conjugated secondary antibody (Biodragon, BF03008) for 1 h at room temperature. Signals were detected using ECL detection reagent (Epizyme, SQ201). The grayscale of the bands in western blotting assay was analyzed by ImageJ software, dividing the grayscale values of the target protein bands from those of the internal reference bands, Obtain the relative intensity of the target protein. All experiments were repeated in three times with duplicate samples.

### RT-qPCR

Total RNA was extracted from the tissue samples using TRIzol^™^ Reagent (Solarbio, 15596-018) and purified by phenol-chloroform extraction (P.C.I). RNA concentration and purity were measured using Nanodrop 2000, and RNA integrity was assessed by agarose gel electrophoresis. cDNA was synthesized using the StarScript III RT Kit (GenStar, A232-10), according to the manufacturer’s instructions. Quantitative PCR was performed using TB Green^®^ Premix Ex Taq^™^ II (Tli RNaseH Plus) (TaKaRa, RR820A) on an Applied Biosystems QuantStudio system, with specific primers for the envelope gene and the housekeeping gene (GAPDH). The thermal cycling conditions were as follows: 95 °C for 10 min, followed by 40 cycles of 95 °C for 15 s, and 60 °C for 1 min. Gene expression was calculated using the ∆∆Ct method and normalized to that of the housekeeping gene (GAPDH). The sequences of the ZIKV Envelope primers were as follows: sense, 5′-CAATCAAGTCCTAGGCTTCCA-3′, and antisense, 5′-ATCCAGCCAGAGAATCTGGAGT-3′. The sequences of the GAPDH primers were as follows: sense, 5′-AGAAGGCTGGGGCTCATTTG-3′ and antisense, 5′-AGGGGCCATCCACAGTCTTC-3′. The following amplification program was used: reverse transcription at 42 °C for 5 min with incubation at 95 °C for 10 s, followed by 40 cycles of 95 °C for 5 s and 60 °C for 20 s. Information collection and melt curve analysis were performed according to the instrument’s manual. All experiments were repeated in three times with duplicate samples.

### Immunofluorescence assay

Immunofluorescence staining was performed to visualize the localization of the envelope proteins. Cells were fixed in 4% paraformaldehyde for 15 min, followed by permeabilization with 0.1% Triton X-100 in PBS for 10 min. After blocking with 5% BSA in PBS for 1 h at 37 °C, following incubation with the primary antibody, cells were washed with PBS and then incubated with Alexa Fluor-conjugated secondary antibody for 1 h at room temperature. The nuclei were stained with DAPI for 10 min at room temperature. Images were captured using a fluorescence microscope. To detect the expression of ZIKV E protein, cells were incubated with an anti-E monoclonal antibody (Clone D1-4G2-4-15, Merck, MAB10216-I) overnight at 4 °C. After washing with PBS for 5 times, the cells were incubated with Alexa Fluor^®^ 488 goat anti-mouse secondary antibody (ab150113, 1:1,000 dilution) for 1 h at room temperature. DAPI (1 μg/mL) was added to stain nuclei. The resulting fluorescence was detected using a confocal microscope (Zeiss LSM 710, Germany). All experiments were repeated in three times with duplicate samples.

### Statistical analysis

Statistical analyses were performed using the GraphPad Prism 9.5. Differences were analyzed using Student’s unpaired *t*-test or one-way ANOVA with Tukey’s multiple comparison test. The data are presented as mean ± SD. Statistical significance was set at *p* < 0.05.

## Data Availability

The RNA-seq data presented in the study are deposited in the NCBI website repository (https://www.ncbi.nlm.nih.gov/sra), accession number is PRJNA1304016. Other data that support the findings of this study are available from the corresponding author [JT] upon reasonable request.
